# The Critical Role of APOE+ Macrophages in the Immune Microenvironment and Prognosis of Lung Adenocarcinoma

**DOI:** 10.1111/jcmm.70731

**Published:** 2025-07-30

**Authors:** Xiaofei Wang, Pengpeng Zhang, Wei Ye, Mingjun Du, Chenjun Huang, Jianan Zheng

**Affiliations:** ^1^ Department of Lung Cancer, Tianjin Lung Cancer Center, National Clinical Research Center for Cancer, Key Laboratory of Cancer Prevention and Therapy, Tianjin's Clinical Research Center for Cancer Tianjin Medical University Cancer Institute and Hospital Tianjin China; ^2^ Department of Thoracic Surgery The First Affiliated Hospital with Nanjing Medical University Nanjing China

**Keywords:** APOE+ macrophage, immune cell infiltration, immune microenvironment, lung adenocarcinoma, risk model

## Abstract

The immunoregulatory functions and clinical implications of APOE+ macrophages within the tumour microenvironment of lung adenocarcinoma remain incompletely defined. In this study, single‐cell transcriptome analysis revealed distinct subsets of APOE+ macrophages, and subsequent CellChat analyses highlighted that these cells predominantly interact with other components of the tumour microenvironment via MIF‐(CD74+CXCR4) and MIF‐(CD74+CD44) signalling pathways, thereby contributing to the establishment of an immunosuppressive milieu. Integrating mutation profiles with multiple machine learning techniques, we developed an APOE+ Macrophage‐related Risk Model (ARM) through a combination of RSF and Ridge approaches, achieving the highest prognostic accuracy (C‐index) among all tested algorithms. The robustness and predictive value of the ARM model were validated across seven public cohorts and three prospective immunotherapy cohorts. Patients classified in the low‐ARM group consistently exhibited better prognoses, as demonstrated by survival analyses, ROC curves and PCA discrimination. Additionally, multiplex immunofluorescence analysis in the in‐house cohort confirmed significantly decreased infiltration of CD4+, CD8+ and CD20+ immune cells in the high‐ARM group, further supporting a more pronounced immunosuppressive microenvironment in these patients. Collectively, our findings not only clarify the critical role of APOE+ macrophages in shaping the immune landscape and prognosis of lung adenocarcinoma, but also provide a validated, practical risk model for individualised patient prognostication and clinical management.

## 1 Introduction

Lung cancer is one of the most prevalent and deadly malignancies worldwide, with lung adenocarcinoma (LUAD) being its major histological subtype and showing a rising incidence in recent years [1, 2]. Although the optimisation of molecular targeted therapy and chemotherapy regimens has led to survival benefits in some LUAD patients, challenges such as recurrence and drug resistance remain severe, and the overall 5‐year survival rate is still low [3, 4]. Tumour immunotherapy, especially the emerging immune checkpoint inhibitors (ICIs) in recent years, has brought new hope for the treatment of LUAD. By releasing T‐cell immune suppression and activating antitumour immunity, ICIs have achieved significant and durable clinical efficacy in some patients with advanced disease [5]. However, a large proportion of patients show limited response or develop resistance to immunotherapy, indicating that the tumour immune microenvironment (TIME) plays a crucial role in determining the efficacy of immunotherapy [6, 7, 8].

The TIME consists of tumour cells, various immune cells, stromal cells and extracellular matrix molecules [9], among which the type, number and status of immune cells directly determine tumour growth rate, invasiveness and sensitivity to treatment [10, 11]. Macrophages are among the most abundant immune cells in the TIME. They may exhibit multiple functional states, such as pro‐tumoral (M2 type) or anti‐tumoral (M1 type) phenotypes [12, 13]. Tumour‐associated macrophages (TAMs) often make up a considerable proportion of immune cells in tumour tissues and regulate tumour growth, metastasis and immune evasion [14]. In recent years, high‐throughput technologies such as single‐cell and spatial transcriptomics have promoted in‐depth analysis of the heterogeneity of macrophages [15], revealing that TAMs have complex subtypes and diverse functions in different tumours or microenvironments, but the precise regulatory mechanisms of specific subpopulations (such as APOE+ macrophages) need further clarification.

Apolipoprotein E (APOE), a gene involved in metabolism and immune regulation, is highly expressed in a macrophage subpopulation (APOE+ macrophages) that has been reported in multiple cancers and is associated with immunosuppression and enhanced invasiveness [16]. Some studies have found that APOE+ macrophages can secrete immunosuppressive factors and participate in signalling pathways to suppress T‐cell activation and promote tumour progression, but their specific roles, distribution features and prognostic relevance in the immune microenvironment of LUAD still require systematic research and validation [16].

Therefore, comprehensively revealing the biological functions of APOE+ macrophages in the LUAD microenvironment, as well as their predictive value for immunotherapy sensitivity and patient prognosis, is of great significance for understanding the differences in immunotherapy response, optimising risk stratification and developing personalised immune intervention strategies. In this study, we aim to elucidate the distribution and signalling networks of APOE+ macrophages in LUAD at the single‐cell level, explore their underlying immunoregulatory mechanisms, and evaluate their associations with tumour prognosis and immunotherapy efficacy, thereby providing a theoretical foundation and novel biological targets for precision immunotherapy in LUAD.

## 2 Method

### 2.1 Data Acquisition and Preprocessing

Gene expression profiles, somatic single nucleotide variants (SNVs) and copy number alteration (CNA) data for LUAD were obtained from The Cancer Genome Atlas (TCGA), while normal lung transcriptome data from the Genotype‐Tissue Expression (GTEx) project served as controls. Chromosomal amplifications and deletions were analysed in the TCGA CNA dataset using the GISTIC 2.0 algorithm. For external validation, six independent LUAD cohorts were downloaded from the Gene Expression Omnibus (GEO): GSE13213 [17] (*n* = 117), GSE26939 [18] (*n* = 115), GSE29016 [19] (*n* = 39), GSE30219 [20] (*n* = 85), GSE31210 [21] (*n* = 226) and GSE42127 [22] (*n* = 133), comprising a total of 715 cases. Batch effects among these datasets were mitigated using ComBat normalisation [23], followed by standard preprocessing. To further evaluate the model within an immunotherapy setting, two LUAD immunotherapy cohorts—POPLAR [24] (*n* = 59) and OAK [24] (*n* = 257)—as well as the SU2C cohort [25] (*n* = 130) comprising NSCLC patients were integrated into the analysis.

### 2.2 Single‐Cell Transcriptomic Analysis

Single‐cell transcriptomic data were obtained from the Genome Sequence Archive (GSA), accession HRA001130. Data preprocessing and normalisation were performed using the Seurat R package (version 4.2.0) [26]. Only genes detected in at least 10 cells per sample were retained for downstream analysis. Stringent cell quality control was conducted by excluding those with gene counts exceeding 5000 or below 200, as well as cells with over 10% of unique molecular identifiers (UMIs) mapped to mitochondrial genes. To address inter‐sample variation, batch correction and dataset harmonisation were carried out using the harmony R package. Highly variable genes were first identified to inform principal component analysis (PCA), and subsequently, the leading 30 principal components were used for t‐distributed stochastic neighbour embedding (t‐SNE) to visualise cellular heterogeneity. Distinct transcriptional signatures for each cell subset were established using the ‘FindAllMarkers’ function, and cell type identification was guided by reference to established canonical markers described in prior studies.

### 2.3 Cell–Cell Communication Analysis

To explore intercellular communication networks within the tumour microenvironment, we employed the CellChat R package [27] to perform cell–cell interaction analysis based on single‐cell RNA sequencing data. After standard normalisation and cell type annotation, the normalised gene expression matrices and cluster identities were used as input for CellChat. The analysis leveraged a curated database of known ligand–receptor pairs to predict potential signalling interactions among annotated cell populations. Communication probabilities were computed under default settings, and the relative contribution of each signalling pathway was quantified.

### 2.4 Identification and Prognostic Modelling of APOE+ Macrophages

Single‐cell transcriptomic data enabled the identification of macrophage subsets characterised by APOE expression. Downstream analyses focused on APOE+ macrophages, utilising their specific molecular markers for further exploration. Differential gene expression was assessed using the limma package [28], with selection criteria of FDR < 0.05 and |log2FC| > 1. Mutational profiles of selected genes were characterised with the maftools package, and visual summaries of frequently altered APOE+ macrophage‐related genes were generated using Oncoplot. For prognostic modelling based on APOE+ macrophage‐specific genes, multiple machine learning algorithms were applied—these machine learning approaches have been previously reported in related research fields [29, 30, 31]. Univariate Cox regression was first used to identify genes significantly associated with patient survival. Ten‐fold cross‐validation was performed to compare and optimise modelling strategies, including stepwise Cox regression, Lasso and Ridge regularisation, CoxBoost, Cox‐adapted partial least squares regression (plsRcox), random survival forest (RSF), gradient boosting machine (GBM), elastic net (Enet), supervised principal components (SuperPC) and survival support vector machines (survival‐SVM). The concordance index (C‐index) served as the primary indicator for model performance evaluation. The final APOE+ macrophage prognostic signature was validated using time‐dependent ROC curves, Kaplan–Meier survival analysis and principal component analysis (PCA).

### 2.5 Immunotherapy Sensitivity and Tumour Immune Microenvironment Analysis

The sensitivity of LUAD patients to immunotherapeutic interventions was evaluated using the immunophenoscore (IPS), derived from data accessed via The Cancer Immunome Atlas (TCIA, https://tcia.at/home) [32]. Further granularity in immune cell composition for TCGA cohorts was achieved utilising TIMER2.0 [33], which aggregates and standardises infiltration estimates from several established analytical methods.

### 2.6 Clinical Specimen Collection and Transcriptome Profiling

Formalin‐fixed, paraffin‐embedded (FFPE) tissue specimens were collected from the Pathology Department at the First Affiliated Hospital of Nanjing Medical University. All samples were histopathologically confirmed as LUAD, and only therapy‐naive patients prior to surgery were included. The study population comprised LUAD patients with complete clinical and follow‐up information who had not received any systemic therapy before surgery. Acquisition of all samples and the conduct of related research were approved by the Institutional Review Board of the First Affiliated Hospital of Nanjing Medical University, with written informed consent obtained from each participant. A portion of the tissue sections was used for multiplex immunohistochemical staining to assess immune cell infiltration within the tumour microenvironment. The remaining tissue specimens were processed for transcriptome sequencing, enabling comprehensive profiling of the molecular characteristics of the tumour samples.

### 2.7 Multiplex Fluorescent Immunohistochemistry Procedure

Multiplex fluorescent immunostaining was performed to assess the association between prognostic scores and immune cell infiltration. Tissue sections were dewaxed in xylene, rehydrated through a series of graded alcohols and subjected to antigen retrieval. Non‐specific binding was blocked with 5% goat serum. The sections were then sequentially incubated with primary antibodies against CD4 (1:500, Cat# ab133616), CD8 (1:2000, Cat# ab217344), CD20 (1:100, Cat# ab64088) and pan‐cytokeratin (panCK, 1 μg/mL, Cat# ab7753). After primary antibody incubation, fluorescent‐labelled secondary antibodies were applied. Cell nuclei were counterstained with DAPI before imaging.

### 2.8 Statistical Methods

All statistical analyses were performed using R software (version 4.2.0). Differences between the two groups were evaluated by unpaired Student's *t*‐test for normally distributed variables, or by the Mann–Whitney *U* test for non‐parametric data. For comparison across multiple groups, one‐way ANOVA with Tukey's post hoc test was used for parametric data, while the Kruskal–Wallis test followed by Dunn's multiple comparison test was used for non‐parametric distributions. Pearson's correlation coefficients quantified relationships between variables. Data are reported as mean ± standard deviation (SD), with *p* < 0.05 considered statistically significant (*p < 0.05, **p < 0.01, ***p < 0.001).

## 3 Result

### 3.1 Single‐Cell Transcriptomic Landscape and Cell Type Identification

**FIGURE 1:**
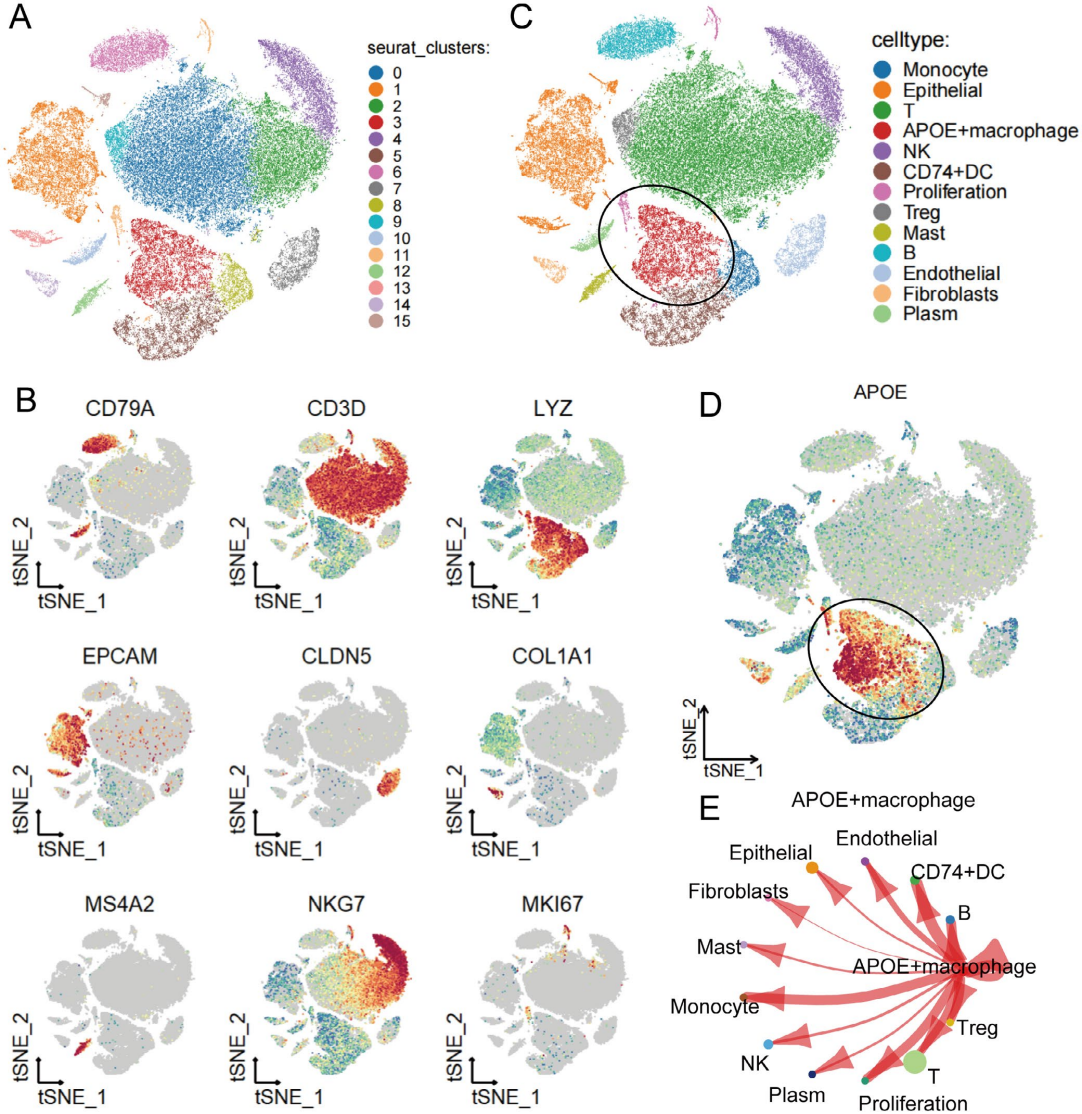
Single‐cell transcriptomic profiling defines cellular heterogeneity and lineage‐specific marker expression. (A) t‐SNE plot showing unsupervised clustering of single‐cell transcriptomes, revealing 16 distinct cell clusters. (B) t‐SNE plots depicting the expression of representative marker genes for major cell types: CD79A (B cells), CD3D (T cells), LYZ (myeloid cells), EPCAM (epithelial cells), CLDN5 (endothelial cells), COL1A1 (fibroblasts), MS4A2 (mast cells), NKG7 (NK cells) and MKI67 (proliferating cells). (C) t‐SNE plot with clusters annotated by cell type, highlighting the APOE+ macrophage population (circled). (D) t‐SNE feature plot showing specific APOE expression in the APOE+ macrophage cluster (circled). (E) Network diagram illustrating cell–cell communication, with arrows indicating interactions between APOE+ macrophages and other cell types.

To comprehensively characterise the cellular heterogeneity of the LUAD tumour microenvironment, we performed single‐cell RNA sequencing (scRNA‐seq) analysis and subsequent unsupervised clustering. t‐SNE dimensionality reduction was applied, which revealed 16 distinct cell clusters within the combined dataset (Figure 1A). Further annotation of these clusters using canonical marker genes enabled the identification of various immune and stromal cell types. The t‐SNE plots of representative marker gene expression (Figure 1B), including CD79A (B cells), CD3D (T cells), LYZ (myeloid cells), EPCAM (epithelial cells), CLDN5 (endothelial cells), COL1A1 (fibroblasts), MS4A2 (mast cells), NKG7 (NK cells) and MKI67 (proliferating cells), confirmed the presence and spatial distribution of these subsets within the tumour microenvironment. Based on these marker profiles, clusters were assigned to major cell types (Figure 1C). Notably, the APOE+ macrophage subset emerged as a distinct population, prominently expressing the APOE marker and highlighted with a black circle in Figure 1D. This cell population has been extensively described in previous studies, with APOE consistently recognised as its characteristic signature gene [16]. To further investigate intercellular interactions, we constructed a cell–cell communication network (Figure 1E), which demonstrated extensive crosstalk between APOE+ macrophages and other cell types, including epithelial cells, T cells, B cells and dendritic cells. These findings illustrate the complex cellular ecosystem in LUAD and specifically highlight the potential regulatory role of APOE+ macrophages within the tumour microenvironment.

### 3.2 APOE+ Macrophage‐Mediated Immune Interactions and Mutation Landscape

**FIGURE 2:**
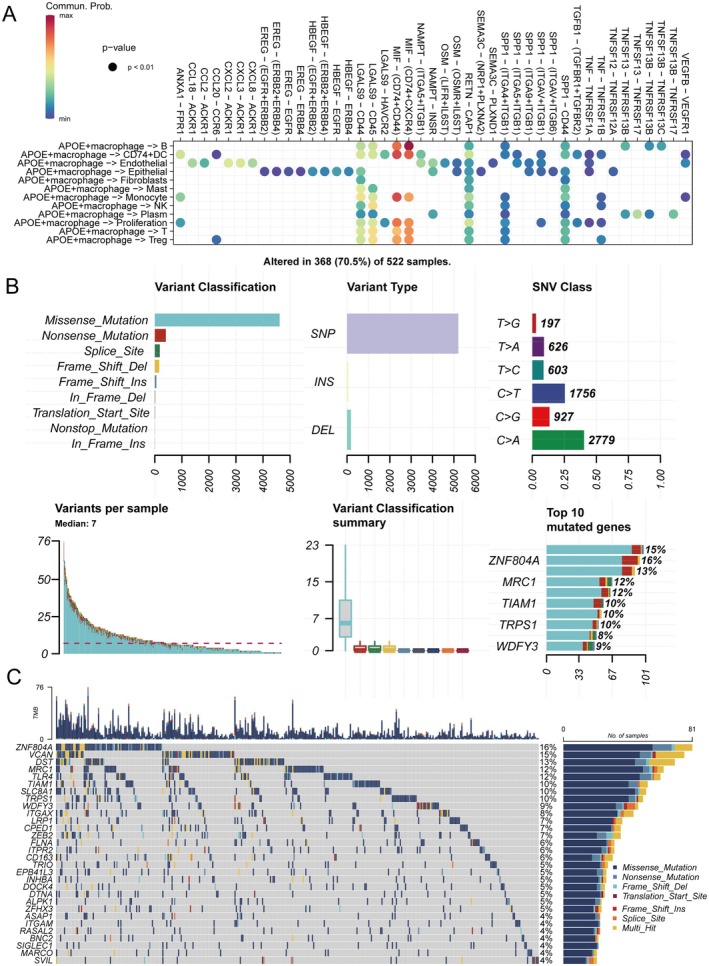
APOE+ macrophage–mediated cell–cell interactions and mutational characteristics in LUAD. (A) Bubble plot illustrating ligand‐receptor interaction probabilities between APOE+ macrophages and other cell types. MIF–(CD74+CXCR4) and MIF–(CD74+CD44) axes, key immunosuppressive pathways, are highlighted in the crosstalk with B cells, DC, monocytes, proliferating cells and Tregs. (B) Summary of variant classification, type and SNV class in APOE+ macrophage marker genes; mutation frequency, variants/sample distribution and top 10 most mutated genes. (C) Waterfall plot of the top 30 most frequently mutated APOE+ macrophage marker genes in the LUAD cohort by sample.

To systematically dissect the intercellular communication patterns of APOE+ macrophages within the LUAD microenvironment, we analysed ligand‐receptor interactions between APOE+ macrophages and other major cell types (Figure 2A). We observed strong interaction probabilities notably involving macrophage‐derived MIF signalling to the corresponding CD74+CXCR4 and CD74+CD44 receptor complexes on B cells, dendritic cells (DC), monocytes, proliferating cells and regulatory T cells (Treg). Previous studies have highlighted MIF as a key immunosuppressive pathway in the tumour microenvironment, reinforcing the significance of these identified interactions [34, 35]. Additional ligand‐receptor pairs, including those mediating crosstalk with epithelial, endothelial and stromal populations, further reveal the central regulatory role of APOE+ macrophages in orchestrating immune cell communication and shaping the immunosuppressive milieu. To further quantify the global cellular interaction landscape, we constructed comprehensive networks displaying both the number (Figure S1A) and strength (Figure S1B) of interactions among all identified cell types. APOE+ macrophages stood out as a key hub with substantial intercellular connections, both in interaction frequency and magnitude, particularly with B cells, T cells and various immune and stromal populations. These integrative results further emphasise the extensive involvement of APOE+ macrophages in modulating the complexity of the tumour immune microenvironment. We next characterised the somatic mutational landscape of APOE+ macrophage marker genes in LUAD samples (Figure 2B,C). Missense mutations represented the predominant variant classification, with single nucleotide polymorphisms (SNPs) comprising the majority of variant types. Among single nucleotide variants (SNVs), C > A and C > T substitutions were most frequently observed, emphasising the mutation spectrum characteristic of these genes. Across samples, a median of seven variants per patient was detected. The top 3 most frequently mutated APOE+ macrophage marker genes included ZNF804A, MRC1 and TIAM1, each harboured in over 10% of samples. Waterfall plot analysis illustrated the mutation status of the top 30 most mutated marker genes, with different mutation types highlighted in the cohort (Figure 2C). Overall, 70.5% (368/522) of samples exhibited at least one alteration in the APOE+ macrophage marker gene set, underscoring their potential importance in tumorigenesis and immune modulation.

### 3.3 Expression Patterns, Genomic Landscape, Prognostic Implications and Functional Enrichment

**FIGURE 3:**
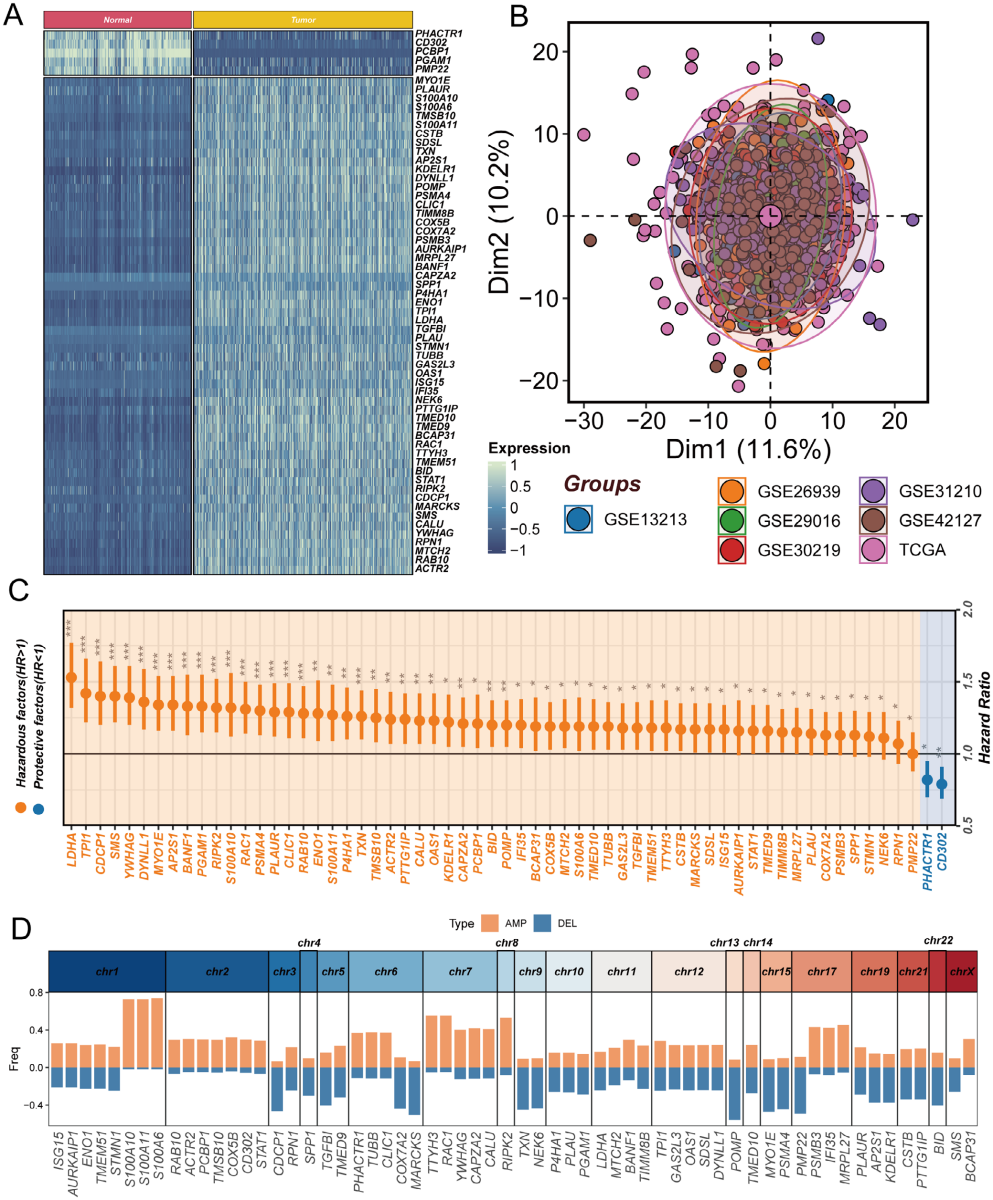
Transcriptomic, prognostic and genomic landscape of APOE+ macrophage marker genes. (A) Heatmap showing differential expression of APOE+ macrophage marker genes between TCGA tumour samples and GTEx normal lung tissues. (B) Principal component analysis (PCA) demonstrating clustering of samples from seven LUAD cohorts after batch effect correction, indicating consistent expression patterns. (C) Forest plot of univariate Cox regression analysis displaying hazard ratios and 95% confidence intervals for differentially expressed marker genes; most genes, including LDHA, TPI1 and CDCP1, are associated with high risk (HR > 1), while PHACTR1 and CD302 are protective genes (HR < 1). (D) Chromosomal distribution and copy number variation (CNV) analysis of marker genes, illustrating regions of amplification (AMP) and deletion (DEL) across the genome.

To further investigate the transcriptional and genomic alterations of APOE+ macrophage marker genes in LUAD, we first compared their expression profiles between TCGA tumour samples and GTEx normal lung tissues. The heatmap analysis revealed widespread and consistent upregulation of multiple marker genes in tumours compared to normal tissues, demonstrating significant tumour‐specific expression patterns (Figure 3A). We then systematically mapped the chromosomal locations of these differentially expressed genes, showing that they are widely distributed across multiple chromosomes (Figure S2A). Red dots represent genes highly expressed in tumours, while black dots indicate genes more highly expressed in normal tissues, underscoring the tissue specificity of their dysregulation. Principal component analysis (PCA) across seven LUAD datasets after batch effect correction showed tight clustering of samples from different cohorts, indicating strong consistency in the expression profiles of these marker genes (Figure 3B). To assess the clinical relevance, univariate Cox regression analysis was performed. The results revealed that the vast majority of differentially expressed marker genes, including LDHA, TPI1 and CDCP1, were consistently associated with increased hazard ratios (HR > 1), identifying them as high‐risk genes linked to poor prognosis (Figure 3C). In contrast, PHACTR1 and CD302 were identified as low‐risk, protective genes (HR < 1), whose high expression predicted a better prognosis. Further, analysis of copy number variation (CNV) and chromosomal localisation demonstrated that many marker genes experienced notable amplifications and deletions across various chromosomes (Figure 3D), suggesting a genomic basis for their aberrant expression. To elucidate the biological functions linked to these changes, KEGG pathway and GO enrichment analysis were performed for the differentially expressed marker genes (Figure S2B). The results showed that these genes were primarily enriched in immune‐related and inflammatory pathways (such as NOD‐like receptor signalling pathway and viral infection), as well as processes related to cytoskeletal organisation and metabolic regulation. This indicates that the dysregulation of APOE+ macrophage marker genes may participate in tumour immune modulation, cellular structural maintenance and stress response mechanisms in LUAD.

### 3.4 Establishment and Validation of the APOE+ Macrophage–Related Prognostic Model

**FIGURE 4:**
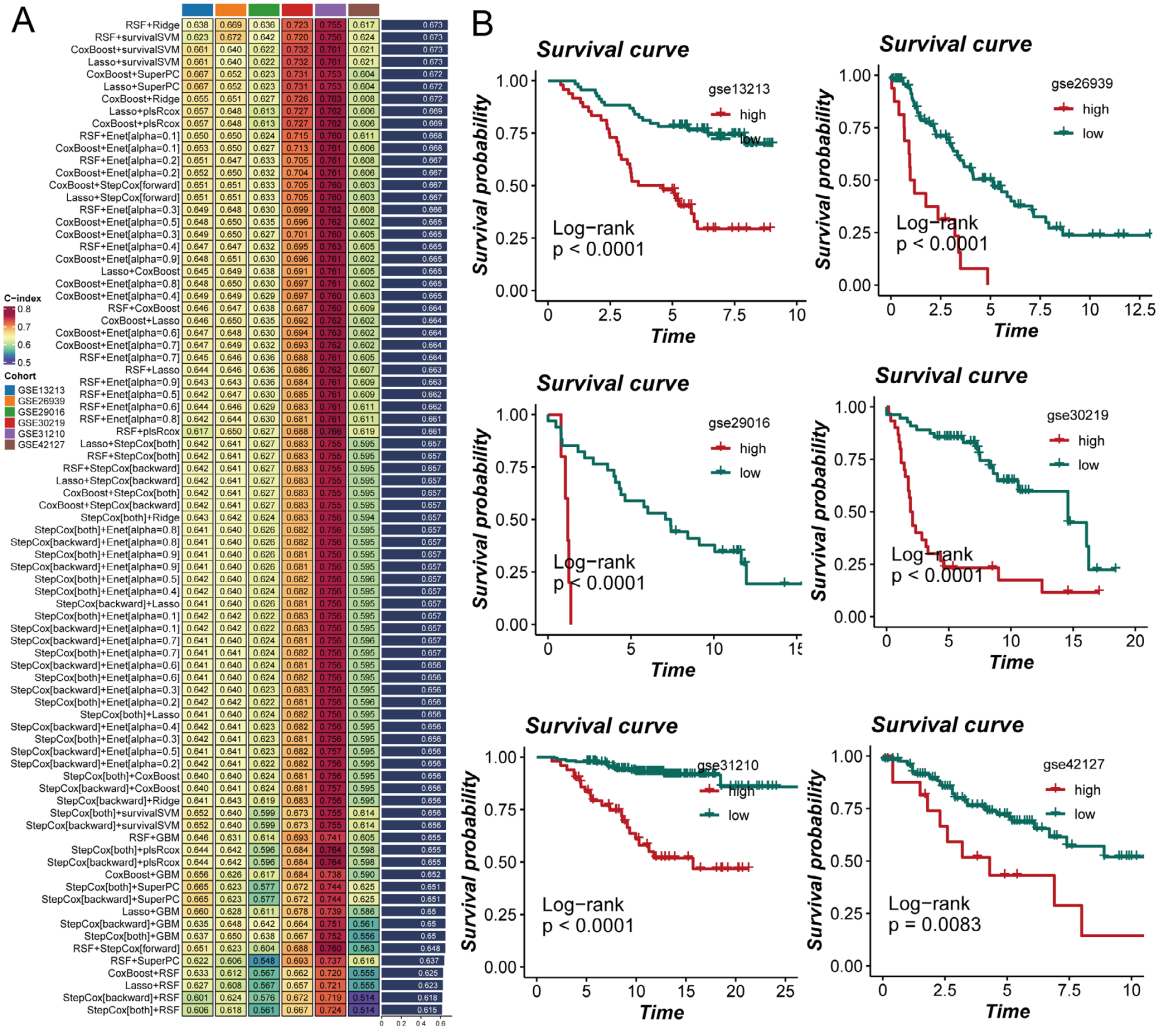
Performance and cross‐cohort validation of the APOE+ macrophage–related risk model (ARM) in LUAD. (A) C‐index heatmap of various machine learning algorithm combinations for prognostic modelling in the TCGA cohort (training) and six external GEO validation datasets. The RSF+Ridge model yielded the highest C‐index and was defined as the ARM. (B) Kaplan–Meier survival curves for the ARM in six GEO validation cohorts. In all cohorts, the high‐risk group experienced significantly worse survival compared to the low‐risk group (all log‐rank *p* < 0.01).

To further evaluate the prognostic value of APOE+ macrophage–related marker genes, we systematically constructed and compared multiple machine learning models based on the TCGA cohort as the training set, with six independent GEO datasets serving as external validation cohorts. Among all algorithm combinations, the RSF+Ridge approach achieved the highest average C‐index and was selected as the optimal predictive model, termed the APOE+ Macrophage–related risk model (ARM) (Figure 4A). Survival analysis using the ARM model in the TCGA training cohort demonstrated that patients classified as high‐risk had significantly worse overall survival compared to those in the low‐risk group (Figure S3A, log‐rank *p* < 0.0001), confirming the predictive value of the model in the training set. Subsequent validation in six independent GEO cohorts further verified the robustness and generalisability of ARM, with all external datasets showing that high ARM scores were consistently associated with poor prognosis (Figure 4B, all log‐rank *p* < 0.01). These findings underscore the strong and stable prognostic power of the ARM model in LUAD.

### 3.5 External Validation of ARM Model Discriminative and Predictive Power

**FIGURE 5:**
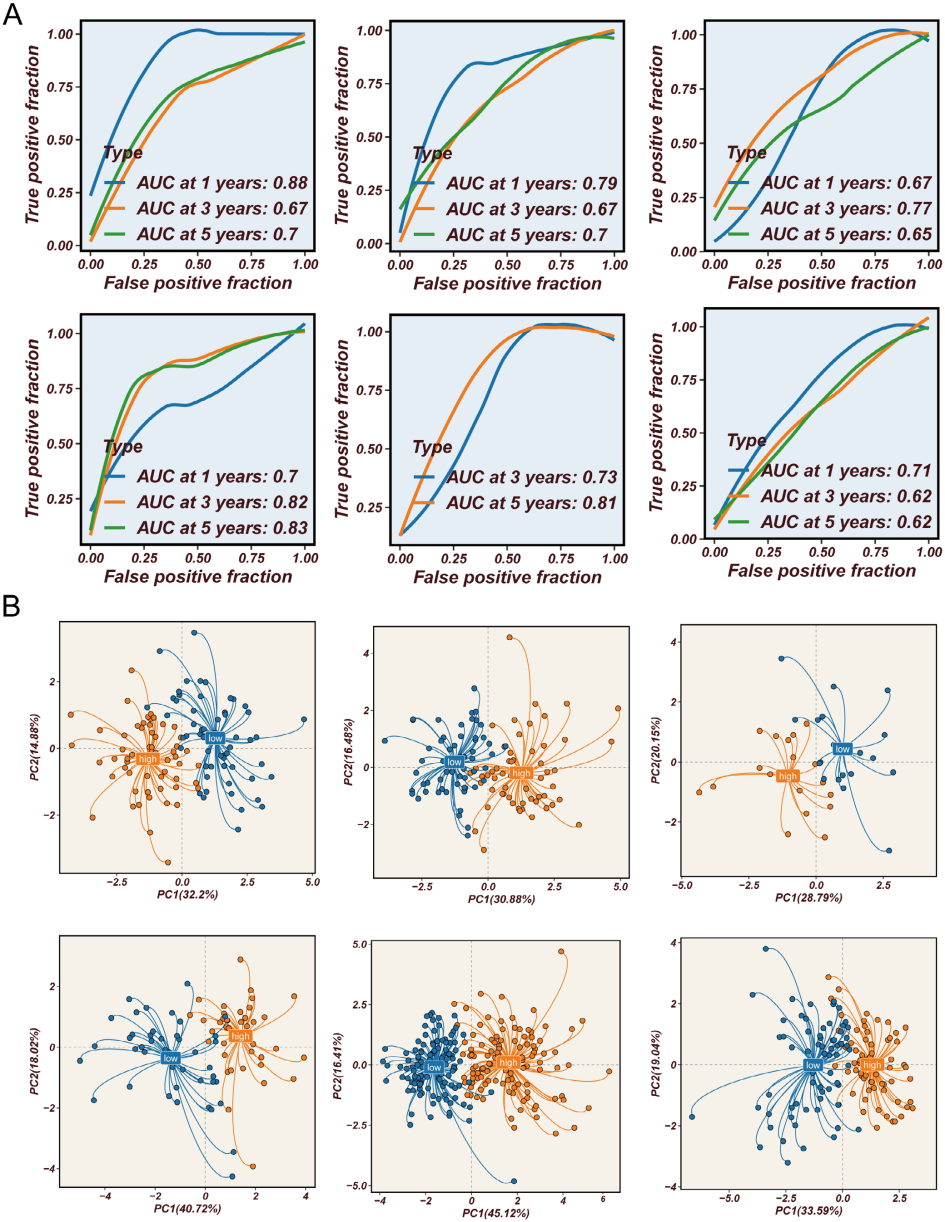
External cohort validation of the discrimination and predictive accuracy of the ARM model. (A) Time‐dependent ROC curves for 1‐, 3‐ and 5‐year overall survival, assessing the predictive power of the ARM across six independent GEO validation datasets. (B) Principal component analysis (PCA) scatter plots illustrating clear separation of high‐risk and low‐risk patients by ARM model genes in each external cohort.

To further validate the clinical application value of the ARM, we systematically evaluated its performance in six independent GEO validation cohorts. Time‐dependent ROC curve analysis showed that the ARM achieved favourable predictive accuracy for 1‐, 3‐ and 5‐year overall survival in these external datasets (Figure 5A), with AUC values largely exceeding 0.7, demonstrating robust and stable prognostic power. Furthermore, principal component analysis (PCA) based on ARM genes effectively separated patients with high and low ARM scores into two distinct clusters in all cohorts (Figure 5B), clearly reflecting the model's potent discriminative ability in external validations. Results from the TCGA training cohort, including time‐dependent ROC curves and PCA plots, are presented in Figure S3B,C, respectively.

### 3.6 The ARM Model Predicts Survival Outcomes in Multiple Public Lung Cancer Immunotherapy Cohorts

**FIGURE 6:**
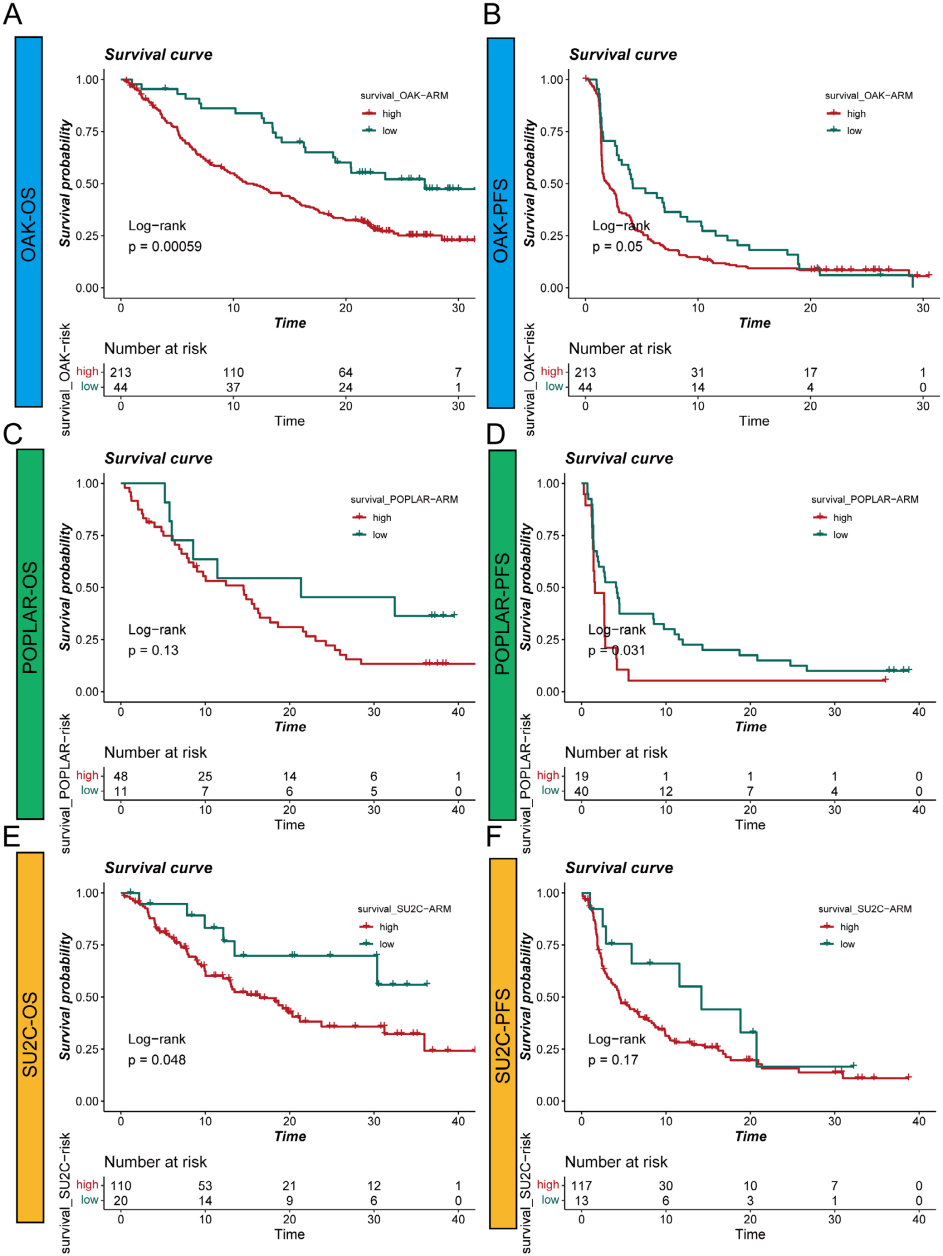
Prognostic value of the ARM model in three public lung cancer immunotherapy cohorts. (A, B) Kaplan–Meier curves illustrating overall survival (OS) (A) and progression‐free survival (PFS) (B) stratified by the ARM risk groups in the OAK cohort. (C, D) OS (C) and PFS (D) curves for the high‐ and low‐ARM groups in the POPLAR cohort. (E, F) OS (E) and PFS (F) curves in the SU2C cohort according to ARM stratification. Across all cohorts, patients in the low ARM group consistently exhibited improved survival compared to those in the high ARM group, supporting the utility of the ARM model for risk assessment in patients receiving immunotherapy.

To further evaluate the utility of the ARM in the context of immunotherapy, we validated its prognostic performance in three public lung cancer immunotherapy cohorts: OAK, POPLAR and SU2C. As shown in Figure 6, Kaplan–Meier survival analyses demonstrated that patients classified into the low ARM group showed significantly improved overall survival (OS) and/or progression‐free survival (PFS) compared to those in the high ARM group across these cohorts. Specifically, both OS and PFS were markedly better in the low‐risk group in the OAK cohort (Figure 6A,B), and similar trends were observed in the POPLAR and SU2C cohorts (Figure 6C–F). These results suggest that the ARM model is not only effective in traditional LUAD cohorts but also serves as a robust prognostic tool for predicting survival outcomes in lung cancer patients receiving immunotherapy, with lower ARM scores indicating a more favourable prognosis.

### 3.7 Distinct Immune Microenvironment Profiles Associated With the ARM Risk Groups

**FIGURE 7:**
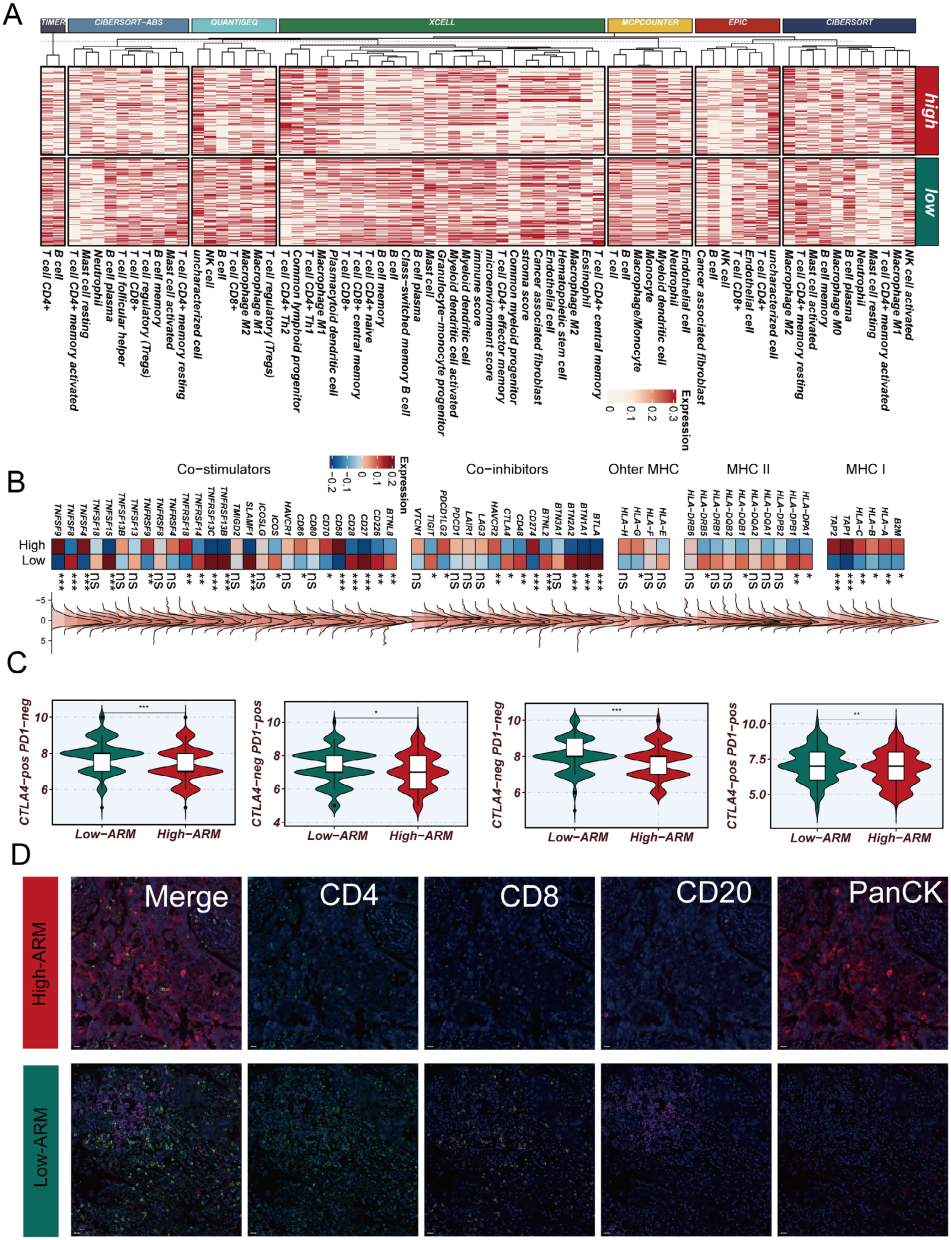
Distinct immune landscapes between the high‐ and low‐ARM groups. (A) Heatmap showing the abundance of tumour‐infiltrating immune cells in the high‐ and low‐ARM groups, estimated by multiple deconvolution algorithms. Low‐ARM tumours had increased infiltration of effector immune cells such as CD4+ T cells, CD8+ T cells and B cells. (B) Expression analysis of immune regulatory molecules, including co‐stimulators, co‐inhibitors and MHC molecules, between the ARM groups. High‐ARM tumours exhibited decreased expression of HLA class II molecules. (C) Immunophenoscore (IPS) comparison between the groups based on TCIA analysis. Low‐ARM tumours displayed higher IPS values, indicating stronger immunogenicity. (D) Multiplex immunofluorescence staining of institutional samples with high and low transcriptomic ARM scores, confirming that high‐ARM tumours were rich in tumour cells (PanCK+) but had fewer infiltrating CD4+, CD8+ and CD20+ immune cells, while low‐ARM tumours showed abundant immune cell infiltration.

To elucidate the immune microenvironmental differences associated with the ARM model, we comprehensively compared the high‐ and low‐risk groups across multiple dimensions. First, immune cell deconvolution analysis using several established algorithms consistently revealed that the low‐ARM group exhibited significantly higher infiltration of key immune effector cells, including CD4+ T cells, CD8+ T cells and B cells (Figure 7A), indicating a more immunologically active tumour microenvironment. Next, gene expression profiling of immune regulatory molecules showed that the high‐ARM group presented with markedly lower levels of HLA class II molecules, as well as certain co‐stimulatory factors (Figure 7B), suggesting impaired antigen presentation and reduced immune recognition in high‐risk tumours. Furthermore, Immunophenoscore (IPS) analysis based on the TCIA database demonstrated that patients in the low‐ARM group had higher IPS values both globally and in specific immune functional subgroups (Figure 7C), supporting the enhanced immunogenicity of these tumours. Finally, multiplex immunofluorescence staining of representative clinical samples, selected based on extreme ARM scores from transcriptomic data, confirmed these findings at the protein level: tumours with high ARM scores showed denser tumour cell (PanCK+) regions but substantially fewer infiltrating CD4+, CD8+ and CD20+ immune cells, whereas low‐ARM tumours were characterised by abundant immune cell infiltration (Figure 7D). Collectively, these results suggest that the ARM model is closely related to the immune microenvironment, with low ARM scores reflecting a more immune‐activated and potentially immunotherapy‐responsive phenotype.

## 4 Discussion

LUAD has long been one of the leading causes of cancer‐related mortality worldwide, with its complex tumour microenvironment (TME) playing a crucial role in tumorigenesis, progression and patient prognosis [36, 37]. In recent years, macrophages—one of the main immune cell components of the TME—have attracted widespread attention due to their diverse functions in regulating immune responses and promoting tumour progression [38, 39, 40]. However, the specific mechanisms and prognostic impact of different functional subpopulations of macrophages, especially those expressing apolipoprotein E (APOE), on the immune microenvironment of LUAD have not been systematically elucidated. Therefore, this study aims to comprehensively explore the immunoregulatory mechanisms of APOE+ macrophages and evaluate their potential as clinical prognostic biomarkers.

Through single‐cell transcriptomic sequencing, this study provides a high‐resolution landscape of the LUAD tumour microenvironment and identifies a distinct subpopulation of APOE+ macrophages characterised by unique transcriptional features. Further cell–cell communication analyses (CellChat) reveal that APOE+ macrophages occupy a central position in the signalling network of the TME, especially engaging in extensive and intimate crosstalk with other major cell types—including T cells, B cells, dendritic cells—via immunosuppressive signalling pathways such as MIF‐(CD74 + CXCR4) and MIF‐(CD74+CD44). These results suggest that APOE+ macrophages play a pivotal regulatory role in tumour immune suppression and microenvironmental remodelling, which is consistent with previous reports regarding the role of the MIF signalling pathway in promoting tumour immune evasion [41, 42].

At the molecular and genomic levels, we found that APOE+ macrophage‐associated marker genes frequently exhibit upregulation, somatic mutations and CNVs in LUAD tissues. Their distinct tumour‐specific expression closely correlates with poor patient prognosis. Through systematic comparison and optimisation of multiple machine learning algorithms, we ultimately established a prognostic risk model (ARM) based on APOE+ macrophage–related genes. This model demonstrated excellent and stable prognostic stratification ability in the TCGA training cohort and six independent GEO external cohorts, with further validation of its strong predictive performance by ROC and PCA analyses. Additionally, the model showed outstanding prognostic value in three prospective immunotherapy cohorts and an internal clinical cohort, with patients in the lower ARM score group exhibiting better overall survival or progression‐free survival. Immunohistochemistry results further confirmed that tumours in the high ARM group displayed significantly reduced infiltration of CD4, CD8 and CD20 cells, indicating a more pronounced immunosuppressive microenvironment.

Further immunological feature analyses revealed that the high ARM group presented diminished antigen presentation capabilities (low HLA molecule expression), impaired activity of key immunoregulatory molecules and immune pathways, while the low ARM group exhibited higher immune cell infiltration and a more active immune phenotype, correlating with better immunotherapy response prediction indicators. These multifaceted results collectively indicate that APOE+ macrophages not only regulate the LUAD immune microenvironment, but also provide vital targets and tools for personalised immunotherapy and risk management.

Despite this study systematically uncovering the central roles of APOE+ macrophages in LUAD immune regulation and prognosis—and establishing a molecular risk model with relatively strong clinical translational potential—there are still some limitations. For example, most of the results are based on multicentre prospective cohorts and retrospective public databases; further prospective validation in real‐world samples is needed. Additionally, more in vivo and in vitro studies are required to further elucidate the functional mechanisms of APOE+ macrophages and their relationship to immunotherapy response.

In summary, this study not only deepens the understanding of the mechanistic roles of APOE+ macrophages within the LUAD tumour immune microenvironment, but also provides innovative theoretical foundations and practical tools for the individualised clinical management and precision immunotherapy of patients with LUAD.

## Author Contributions

**Xiaofei Wang:** data curation (equal), formal analysis (equal), validation (equal). **Pengpeng Zhang:** conceptualization (equal), data curation (equal), validation (equal). **Wei Ye:** data curation (equal), formal analysis (equal), validation (equal). **Mingjun Du:** data curation (equal), formal analysis (equal), supervision (equal). **Chenjun Huang:** conceptualization (equal), data curation (equal), writing – original draft (equal), writing – review and editing (equal). **Jianan Zheng:** data curation (equal), formal analysis (equal), validation (equal), writing – original draft (equal), writing – review and editing (equal).

## Ethics Statement

Our research methodology aligned with Helsinki Declaration standards and was endorsed by the Ethics Board of The First Affiliated Hospital with Nanjing Medical University (Approval No. 2019‐SR‐156). Participants were enrolled only after providing documented informed consent.

## Conflicts of Interest

The authors declare no conflicts of interest.

## Supporting information


**FIGURE S1.** Global cellular crosstalk networks: (A) Number of intercellular interactions among all cell types; (B) Interaction weights/strengths reveal APOE+ macrophages as a key communication hub, especially with B cells, T cells and other immune/stromal cells.

**FIGURE S2.** Chromosomal localisation and functional enrichment of differentially expressed APOE+ macrophage marker genes. (A) Circos plot showing chromosomal positions of marker genes; red dots indicate tumour‐high genes, black dots indicate normal‐high genes. (B) KEGG pathway (left) and GO enrichment (right) for these genes, revealing significant enrichment in immune/inflammatory signalling (e.g., NOD‐like receptor pathway, neurodegeneration, infection) and cytoskeletal/metabolic processes (e.g., actin filament organisation, ubiquitin‐protein ligase binding).

**FIGURE S3.** Prognostic evaluation of the ARM model in the TCGA cohort. (A) Kaplan–Meier survival curves for the high‐ and low‐risk groups. (B) Time‐dependent ROC curves for 1‐, 3‐ and 5‐year survival. (C) PCA plot showing separation of risk groups.

## Data Availability

The data that support the findings of this study are available from multiple public databases and repositories. Single‐cell RNA sequencing data were obtained from the Genome Sequence Archive (GSA) under accession number HRA001130. Bulk RNA sequencing data were accessed from The Cancer Genome Atlas (TCGA‐LUAD, http://cancergenome.nih.gov/) and the Genotype‐Tissue Expression (GTEx) project. Independent validation cohorts were collected from the Gene Expression Omnibus (GEO, https://www.ncbi.nlm.nih.gov/geo/) database (GSE13213, GSE26939, GSE29016, GSE30219, GSE31210 and GSE42127). Immunotherapy cohort data were obtained from previously published studies (POPLAR, OAK and SU2C cohorts). Somatic mutation and CNV data were retrieved from The Cancer Genome Atlas (TCGA). The processed data that support the findings of this study are available from the corresponding author upon reasonable request.
