# Supplementary figures and images for: The Critical Role of APOE+ Macrophages in the Immune Microenvironment and Prognosis of Lung Adenocarcinoma
Source: J Cell Mol Med. 2025 Jul 30;29(15):e70731. doi: 10.1111/jcmm.70731 (PMC12308911; doi:10.1111/jcmm.70731)

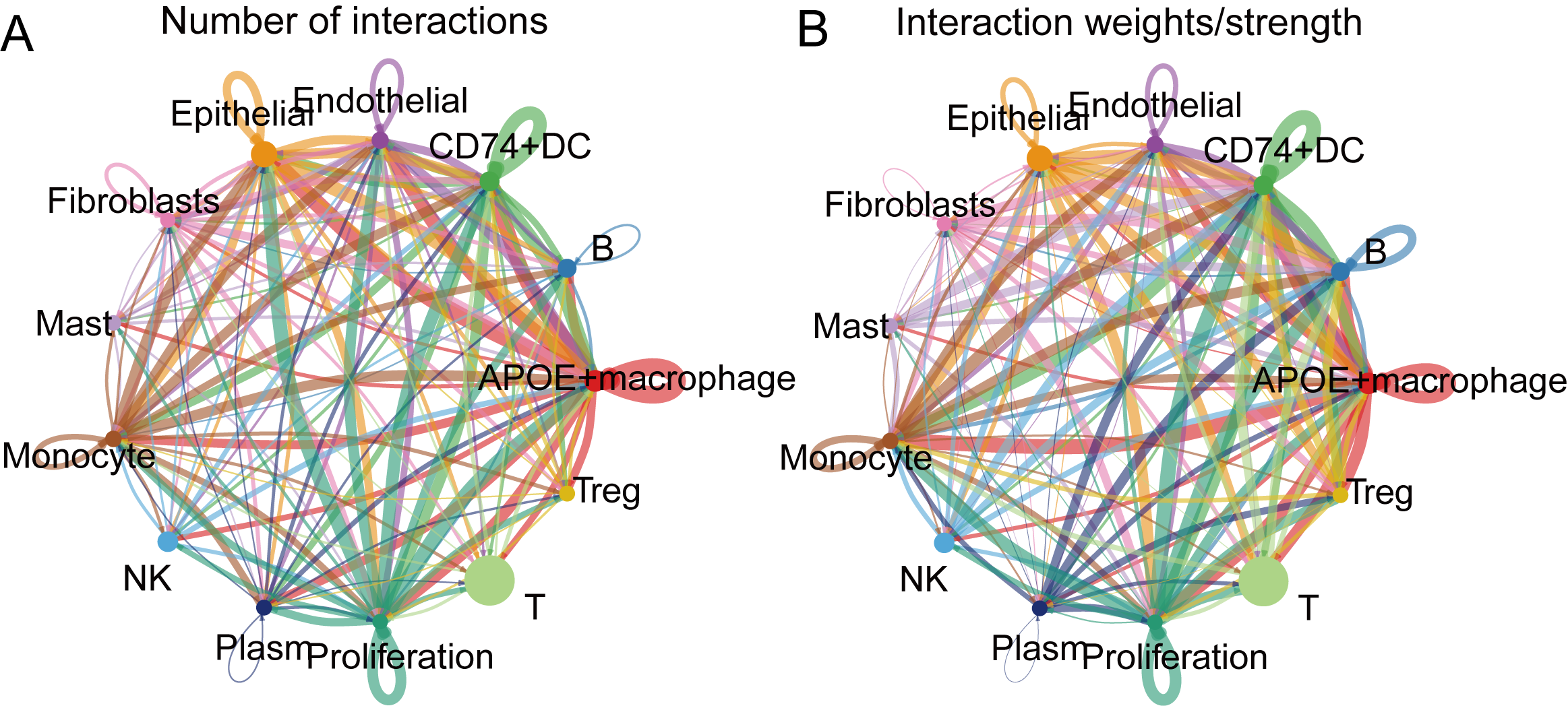

Supplement: Supplementary file 1 — FIGURE S1. Global cellular crosstalk networks: (A) Number of intercellular interactions among all cell types; (B) Interaction weights/strengths reveal APOE+ macrophages as a key communication hub, especially with B cells, T cells and other immune/stromal cells. [file JCMM-29-e70731-s001.tif]

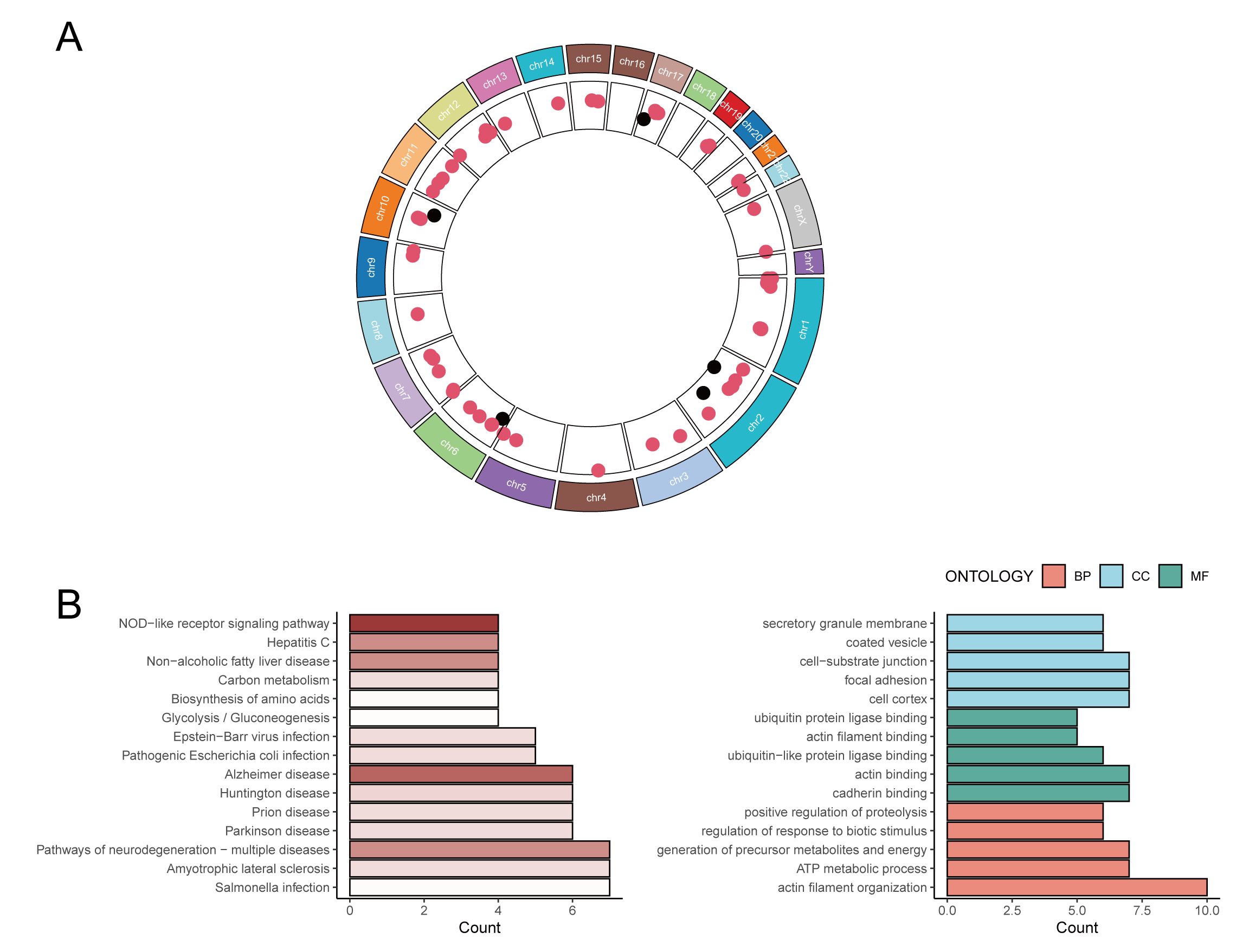

Supplement: Supplementary file 2 — FIGURE S2. Chromosomal localisation and functional enrichment of differentially expressed APOE+ macrophage marker genes. (A) Circos plot showing chromosomal positions of marker genes; red dots indicate tumour‐high genes, black dots indicate normal‐high genes. (B) KEGG pathway (left) and GO enrichment (right) for these genes, revealing significant enrichment in immune/inflammatory signalling (e.g., NOD‐like receptor pathway, neurodegeneration, infection) and cytoskeletal/metabolic processes (e.g., actin filament organisation, ubiquitin‐protein ligase binding). [file JCMM-29-e70731-s002.tif]

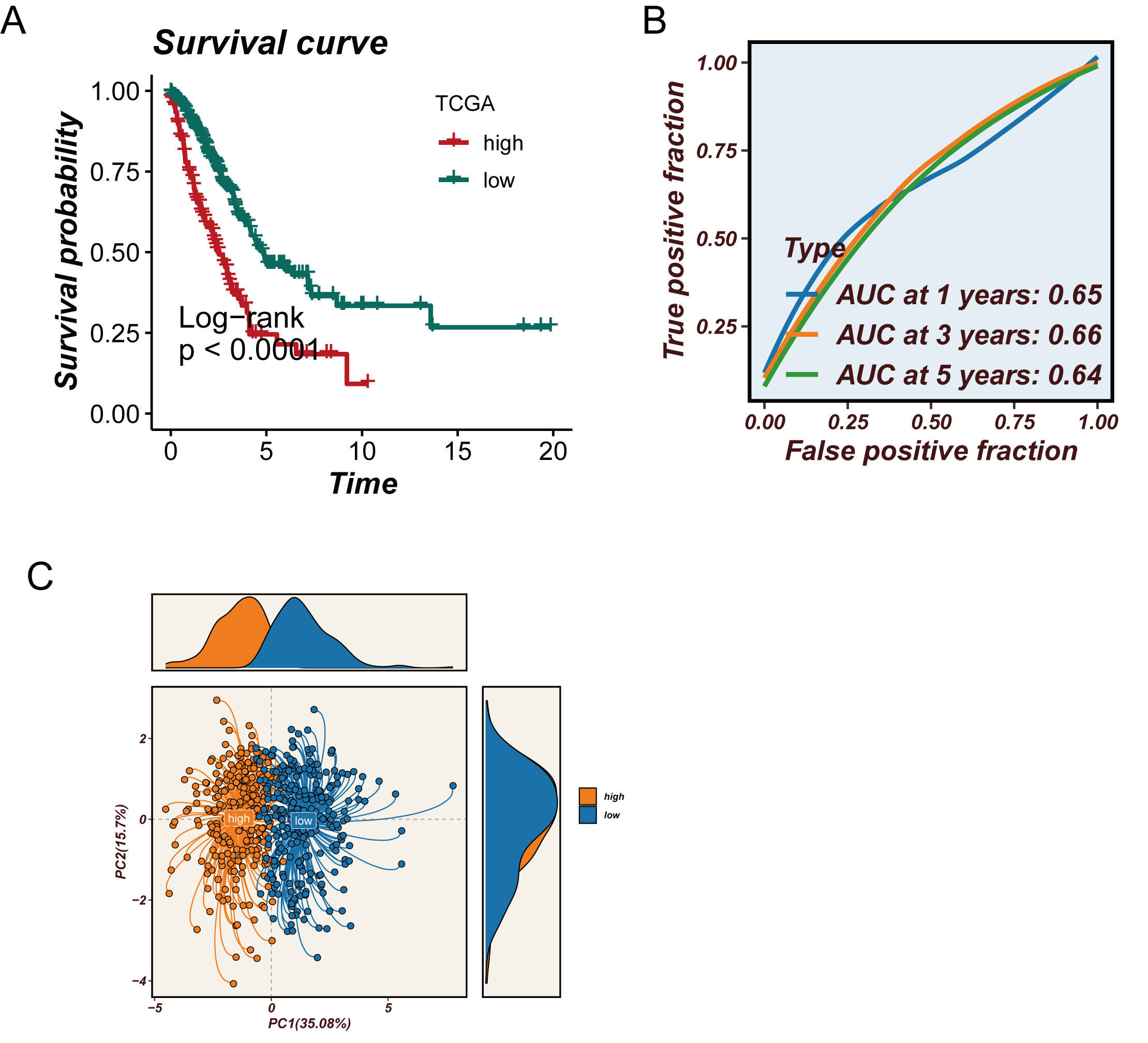

Supplement: Supplementary file 3 — FIGURE S3. Prognostic evaluation of the ARM model in the TCGA cohort. (A) Kaplan–Meier survival curves for the high‐ and low‐risk groups. (B) Time‐dependent ROC curves for 1‐, 3‐ and 5‐year survival. (C) PCA plot showing separation of risk groups. [file JCMM-29-e70731-s003.tif]
